# Wear and Corrosion Resistance of AlSi10Mg–CP–Ti Metal–Metal Composite Materials Produced by Electro-Sinter-Forging

**DOI:** 10.3390/ma14226761

**Published:** 2021-11-10

**Authors:** Federico Simone Gobber, Elisa Fracchia, Roberto Spotorno, Alessandro Fais, Diego Manfredi

**Affiliations:** 1 Department of Applied Science and Technology (DISAT), Polytechnic of Turin, Corso Duca degli Abruzzi 24, 10124 Torino, Italy; diego.manfredi@polito.it; 2 Consorzio Interuniversitario di Scienza e Tecnologia dei Materiali (INSTM), Via G. Giusti 9, 50121 Firenze, Italy; 3 Department of Chemistry and Industrial Chemistry (DCCI), University of Genoa, Via Dodecaneso 31, 16146 Genoa, Italy; roberto.spotorno@unige.it; 4 Epos s.r.l., Via Pavia 68/72, 10098 Rivoli, Italy; af@eposintering.com

**Keywords:** AlSi10Mg, CP–Titanium, electro-sinter-forging, metal–metal composites, electrochemical impedance spectroscopy E.I.S., tribological testing, Raman

## Abstract

Metal–metal composites are a class of composite materials studied for their high ductility and strength, but their potential applications are currently limited by the complex manufacturing processes involved. Electro-sinter-forging (ESF) is a single-pulse electro discharge sintering technique that proved its effectiveness in the rapid sintering of several metals, alloys, and composites. Previous studies proved the processability of Ti and AlSi10Mg by ESF to produce metal–metal composites and defined a correlation between microstructure and processing parameters. This paper presents the wear and corrosion characterizations of two metal–metal composites obtained via ESF with the following compositions: 20% Ti/80% AlSi10Mg and 20% AlSi10Mg/80% Ti. The two materials showed complementary resistance to wear and corrosion. A higher fraction of AlSi10Mg is responsible for forming a protective tribolayer in dry-sliding conditions, while a higher fraction of Titanium confers improved corrosion resistance due to its higher corrosion potential.

## 1. Introduction

Metal–metal composites have gained significant attention in the research for innovative materials, thanks to an improved balance between high strength and ductility [1,2,3]. These composite materials can be adopted to overcome the characteristic drawbacks of metal/ceramic composites: weak interface bonding between the metal and the ceramic phases and low ductility. An additional advantage of exploring these systems is that new and innovative materials may be realized by balancing their properties due to the amount and type of metals involved [4,5].

The combination of the lightweight and relatively low melting point of aluminum has attracted plenty of attention, in the past, as one of the constituents of these novel metal–metal composites. For instance, Madhusudan et al. [1] studied Al–Cu composites with different Cu content (5% to 15%) prepared by stir casting. The work aimed to produce a composite material, Al–Cu, with the same chemical and aging properties as the conventional casting alloy Al–Cu. The results highlight that the composite with 5% wt. of Cu may show the same chemistry as the casting alloy but only 85% of its hardness (92 HV for the aged conventional alloy, 85 HV for the stir cast composite) but a ductility improved by 15%.

In [6], metal–metal composites made in aluminum alloy AA2024 and 316L stainless steel were prepared by mechanical milling and hot pressing. The authors obtained high-performance composites in which the 316L particles resulted well bonded to the aluminum matrix with nucleation of intermetallic Fe–Al phases. The amount of intermetallics increases by increasing 316L (from 10% to 25%). The authors observed an inverse relationship between corrosion resistance and wear resistance as a function of the number of intermetallic compounds: as the number of intermetallics increased, the wear resistance increased while the corrosion resistance decreased. Other studies successfully developed intermetallic compounds between Al and Ti starting from the pure elements or pure Ti and an Al alloy.

The most successful approaches reported in the literature to obtain metal–metal composite materials are those employing the deformation or rolling of thin sheets of ductile metals [7], explosive welding starting from thin foils [8], or cold roll bonding [9]. On the other hand, despite being near-net-shape and rapid, powder metallurgical techniques based on electric field-assisted sintering (e.g., Spark Plasma Sintering) proved ineffective in consolidating powders without avoiding the formation of intermetallic compounds [10,11,12]. Nevertheless, powder metallurgy is widely used to produce Al-based composites. In particular, in [13], authors have explored the mechanical milling followed by a hot pressing process technique to produce homogenous AA7075-0.5 wt% Y_2_O_3_ composites. They found that the increase in ball milling time is helpful to the homogeneous dispersion of Y_2_O_3_; however, ball milling up to two hours increase the particle hardness, reducing the tensile strength.

Physical and mechanical properties in Al-based composites change as a function of the processing parameters adopted. In [14], the authors deduced an optimum set of parameters for producing Al-based composites, considering a compaction pressure ranging between 600–700 MPa and a sintering temperature between 520–600 °C for 3 or 4 h. They furthermore found that the compaction pressure is the most influencing parameter while the maximum fraction of reinforcement microparticles before agglomeration starts is about 10%. Similar behavior was highlighted in [15], where hybrid composites were produced. In this case, the Aluminum powder was mixed with SiC and Al_2_O_3_, finding that an excessive reinforcement addition negatively affects sinterability, density, hardness, and, last but not least, the compressive strength.

Among powder metallurgical techniques, Electro-sinter-forging (ESF) [16,17], a single pulse electro-discharge sintering (EDS) technique [18], was shown to be effective in producing a Ti–AlSi10Mg metal–metal composite [19]. By properly tuning the process parameters, densities up to theoretical ones are achievable without intermetallic phases. In the previous work, two types of metal–metal composites were studied: (i) CP–Ti matrix and AlSi10Mg second phase and (ii) AlSi10Mg as the matrix and CP–Ti as the second phase. The absence of TiAl intermetallics could positively affect corrosion resistance by eliminating sources for pitting. In contrast, the Mg_2_Si intermetallic phase, already present in the starting powders, may still be present in small amounts and could behave as an anodic site for the corrosion of the Al-rich areas [20].

As to wear resistance, the poor tribological properties of Titanium are related to its high strength and low thermal conductivity, even at high temperatures. In [21], the tribological properties of three Ti–6Al–4V alloys were studied under dry sliding conditions against pins made from different steels. Wear developed in the tribological pair is attributed to abrasion and adhesion mechanisms with an increased coefficient of friction with increasing sliding time. Conversely, AlSi10Mg alloy presents good wear resistance, thanks to the possibility of forming a protective tribolayer under specific sliding conditions [22].

This work characterizes the wear and corrosion resistance of two AlSi10Mg/CP–Ti metal–metal composites by pin-on-disc and electrochemical tests. Worn surfaces are further studied by Scanning Electron Microscopy (SEM) and Energy Dispersive Spectroscopy (EDS), while corrosion products were analyzed through Raman spectroscopy.

## 2. Materials and Methods

**Figure 1:**
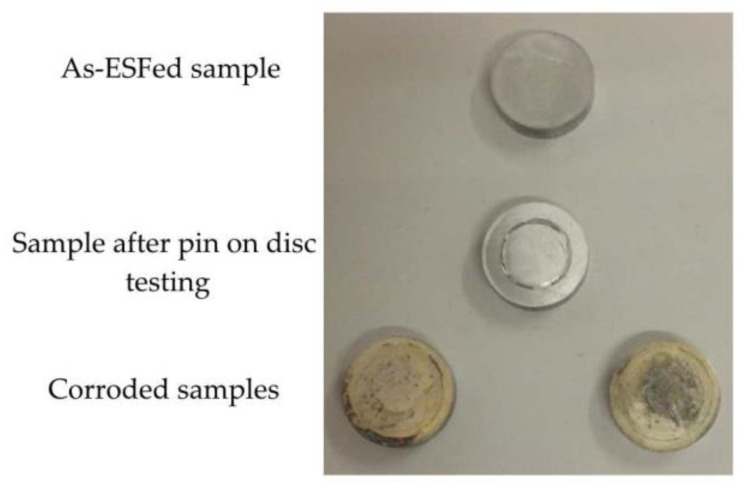
Macrograph of the samples employed for characterizing wear and corrosion resistance.

Commercially pure (CP) Ti-Grade2 powders (Metco 4013A with 45–106 µm PSD and 2.4 g/cm^3^ tap density) and AlSi10Mg powders (Ecka Germany GmbH, Alumix, with 10–160 µm PSD and 1.6 g/cm^3^ tap density) whose composition is reported in Table 1, were manually mixed and then densified via ESF to obtain disc-shaped samples 15 mm in diameter and 5 mm in nominal thickness (Figure 1). The powders were weighted with a Kern PLS 510-3 precision balance and manually loaded through a funnel placed inside a die. The die was previously mounted on a machine for Electro-Sinter-Forging by EPoS equipped with a press able to reach a maximum force of 120 kN and a generator of electric current able to provide up to 100 kJ of energy to the loose powders. The peak pressure (MPa) and the amount of energy charged in the capacitor banks (kJ) discharged on the powders were progressively increased from one sample to the subsequent one up to the theoretical density. A detailed characterization of the influence of the processing parameters on the microstructure is presented in [19].

Two compositions of Ti/AlSi10Mg metal–metal composite materials made by Electro-Sinter-Forging were studied with CP–Ti matrix and AlSi10Mg as the second phase (hereafter named Ti80–AlSi) and a second composition with AlSi10Mg as the matrix and CP–Ti as the second phase (hereafter named Ti20–AlSi). Fully dense samples processed at low energy (low amount of energy discharged on the powders), without any intermetallic phase, were chosen for the characterization. Samples were manufactured at 300 MPa peak pressure and energies as high as 860 J/g (Ti80–AlSi) or 1822 J/g (Ti20–AlSi). Tribological analysis was carried out in sliding conditions, whereas the corrosion resistance was determined in an aerated solution of 3.5% NaCl. A previous study has presented the optimization of the processing route to manufacture such metal–metal composite materials [19], together with a comprehensive microstructural description.

### 2.1. Tribological Testing

Ball-on-disc wear tests were performed at 25 °C and 50% relative humidity according to ASTM G99-17 on a laboratory-built tribometer. The test parameters are reported in Table 2; the number of rotations on the pin on the disc tribometer was set to give a total sliding distance of 100 m. The Hertzian contact pressure was 852 MPa and was comparable to other studies on Al-based metal–matrix composite materials [23]. The stresses due to the non-conformal contact between the two surfaces were calculated using the Hertz contact stress calculator Hertzwin [24]. The program numerically solves the elliptic integrals of the Hertz formulas and returns all the characteristic features of the contact as outputs: contact radius, maximum contact pressure, maximum shear stress, Von Mises Stress. For the calculation, we adopted the ball-on-flat configuration for non-conformal point contact. The input parameters were: finite ball radius, infinite flat surface radius, normal load, surface roughness, Young’s modulus, Poisson’s modulus (0.33), and the friction coefficient derived from the ball on disc test. This approach is proposed to support the morphological, microstructural, and compositional observations: the derived results are intended as a qualitative representation of the stress distribution below the pin-disc contact area. Indeed, as a simplification, due to the complexity of determining Young’s modulus for the two metal–metal composite materials, pure Titanium (102 GPa) was employed when calculating Ti80/AlSi related contact stresses, and pure Aluminum Young’s modulus (70 GPa) was employed to compute Ti20/AlSi related contact stresses.

Three tests for each condition were performed, and their mean value was used to determine the overall friction coefficient. After each test, the hard pin was removed from the holder, brushed slightly, cleaned in an ultrasonic bath with acetone to remove wear debris, dried at 80 °C, and placed back in the holder. The following formula (1) determined the wear rate.

(1) $$\mathrm{Wear}\;\mathrm{rate}\;\left(\frac{mm^{3}}{m\cdot N}\right)=\frac{{\mathrm{Volume}\;\mathrm{loss}\;(\mathrm{mm}}^{3})}{\mathrm{Load}\;(N)\cdot \mathrm{Distance}\;(m)}=10^{3}\cdot \frac{\mathrm{Weight}\;\mathrm{loss}\;(g)}{\mathrm{Density}\;(\frac{g}{{\mathrm{cm}}^{3}})\cdot \mathrm{Load}\;(N)\cdot \mathrm{Distance}\;(m)}$$

The weight loss of the composite samples was measured with 0.1 mg accuracy by weighting the difference before and after the sliding tests. Density values used for calculation are respectively 3.04 g/cm^3^ for Ti20–AlSi and 4.13 g/cm^3^ for Ti80–AlSi.

The wear tracks and pin surfaces were observed from the top by scanning electron microscope (SEM, Zeiss E.V.O. 15, Zeiss, Oberkochen, Germany) equipped with secondary and backscattered electrons detector and analyzed by energy dispersive spectroscopy (EDS, Oxford Ultim Max, Oxford Instruments plc, Abingdon, UK). The metal–metal composite samples were then cut in half, mounted in phenolic resin, and prepared for metallographic observation by grinding up to 1200 grit abrasive paper and subsequently polished on a cloth soaked with colloidal silica.

### 2.2. Electrochemical Characterization

Electrochemical characterization was performed on both Ti80–AlSi and Ti20–AlSi, to evaluate the effect of composition on corrosion resistance, following a procedure detailed in our previous works [25,26].

The tests were carried out in a naturally aerated solution of 3.5% NaCl (99.9%, V.W.R.) in deionized water (pH = 7.4) using a proprietary flat cell with three electrodes designed to expose a fixed round area of the sample (0.5 cm^2^). The cell configuration consisted of a working electrode (WE, sample), a reference electrode of Ag/AgCl sat.KCl (Ref, AMEL, S.r.l., Milano, Italy) and a Pt counter electrode (C.E., AMEL S.r.l, Milano, Italy). Electrochemical measurements were performed using a galvanostat/potentiostat (V.S.P., BioLogic, Seyssinet-Pariset, France). The open-circuit potential (OCP) was recorded for 1 h to reach a steady state before electrochemical characterization. Electrochemical impedance spectroscopy (E.I.S.) measurements were performed with an A.C. amplitude of 10 mV vs. OCP in the frequency range 200kHz–1Hz and a density of 10 points/decade. The E.I.S. spectra were analyzed by equivalent electric circuit (E.E.C.) fitting using the complex nonlinear regression least squares (CNRLS) method performed with ZView^®^ software (Scribner Associates INC., Southern Pines, NC, USA), accepting a fitting error of less than 10% for each circuit element. Polarization curves were measured from −1.0 to −0.4 V vs. Ag/AgCl with a scan rate of 0.5mV s^−1^ and analyzed using the software CView^®^ (Scribner Associates INC., Southern Pines, NC, USA). Solution conductivity and pH were measured before and after each test using a digital pH meter/conductometer (X.S. PC 60 VioLab, Giorgio Bormac S.r.l., Carpi, Italy).

After the electrochemical tests, the corroded microstructures were observed and analyzed by SEM-EDS. Finally, the corrosion products were characterized by Raman spectroscopy (Renishaw, InVia Raman Microscope, Pianezza, Italy) by Ar laser source 514 nm/50mW, 10 accumulations per scan. Low laser power was chosen to avoid damaging the corrosion products.

## 3. Results and Discussion

### 3.1. Tribological Testing

#### 3.1.1. Coefficient of Friction

**Figure 2:**
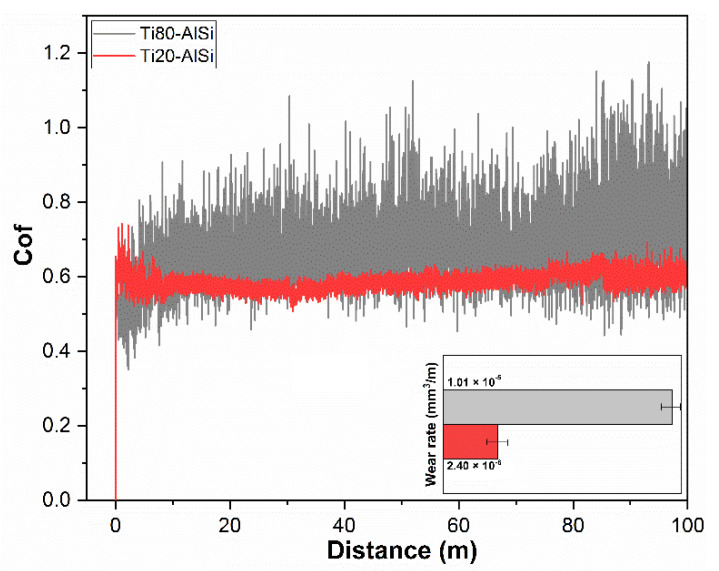
Coefficient of friction and wear rate against a WC–6Co ball for the two materials Ti80–AlSi and Ti20–AlSi.

Although the average coefficient of friction (COF), respectively 0.53 for the Ti20–AlSi and 0.59 for the Ti80–AlSi, is similar, its variations in time/distance, shown in Figure 2, differ significantly. The coefficient of friction for the Ti80–AlSi scatters during the test (0.05 vs. 0.09 standard deviation), and its average value slightly increases over the sliding distance. The Ti80–AlSi curve is characterized by a short initial run-in period followed by large fluctuations. Such scattering reflects the accumulation and detachment of wear debris during the tribological process. Such mechanisms, attributable to adhesion, extrusion, or fragmentation caused by Ti’s continuous transfer to WC–6Co, are described in the literature as a periodic phenomenon formation and pull out of a tribolayer [21]. In other words, what occurs is a sliding wear process never reaching the typical equilibrium conferred by a protective tribolayer.

The COF over distance curve for the Ti20–AlSi sample shows an initial run-in period with a higher COF in which the surface asperities of the WC–6Co/Ti80–AlSi conform to one another, with a predominance of metal sliding against metal. The COF of the Ti20–AlSi sample, however, reaches a stable value over distance. If the wear rate is also considered, the difference between the two materials is clear: the Ti80–AlSi composite has a wear rate that is one order in magnitude higher (1.01 × 10^−5^ vs. 2.4 × 10^−6^ mm^3^/m).

#### 3.1.2. Wear Mechanisms Characterization

**Figure 3:**
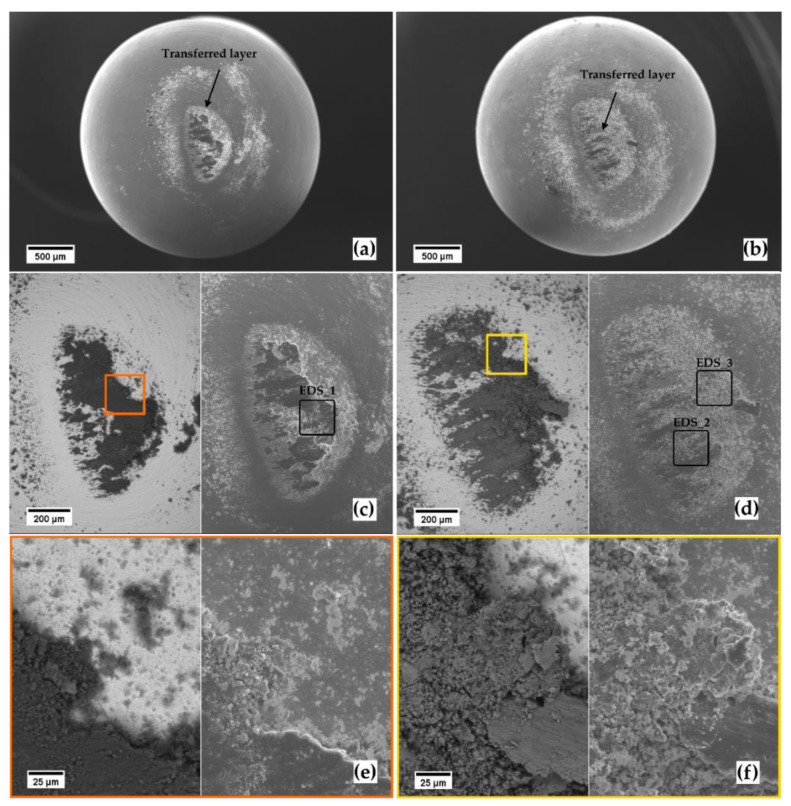
SEM micrographs. Worn ball-end surface at increasing magnification for the Ti20–AlSi (**a**,**c**,**e**), and for the Ti80–AlSi (**b**,**d**,**f**). (**a**,**b**) and the left micrographs of (**c**–**f**) are from the backscattered electrons detector; the secondary electron detector acquired the remaining micrographs.

The morphology of the debris deposited on the WC–6Co ball-end pins surface is presented in Figure 3a,b. Comminuted debris is detected together with a larger central area composed of transferred material; such central area is broader on the pin sliding against the Ti80–AlSi disc (Figure 3a,c), in accordance with the higher wear rate measured on the corresponding disc. As a result, the tribolayer transferred in the Ti20–AlSi/WC–6Co tribopair appears more solid and adhered with signs of plastic deformation (Figure 3c,e) while the transferred layer in the Ti80–AlSi/WC–6Co tribopair is more fragmented and discontinuous (Figure 3d,f). This observation suggests a transferred layer formed in the Ti80–AlSi sample with reduced ductility continuously ground and comminuted in small particles, and cannot form a stable tribolayer.

The AlSi10Mg domains oxidize more readily, as measured by the EDS. As a result, the weight fraction of O in EDS_1, related to Ti20–AlSi, doubles compared to the EDS_2 and EDS_3 regions of Ti80–AlSi. Therefore, the higher quantity of AlSi10Mg in the sample strongly influences the composition of the transferred layer, resulting in increased oxygen content for the Ti20–AlSi related transferred layers (Table 3). The oxidized tribolayer thereby reduces the tribological affinity between the two sliding surfaces by changing the composition of one of them. As a result, following the classical theories of adhesive wear [27], an oxide layer has a lower reactivity towards the parent metal and reduces the tendency to form micro-welding at the contact asperities during sliding, thus restraining the wear rate.

**Figure 4:**
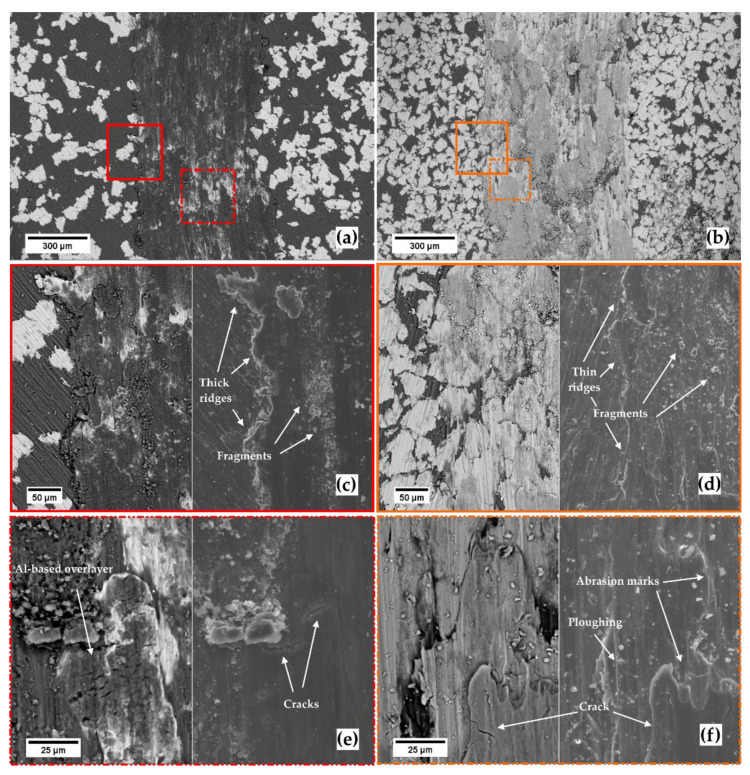
SEM micrographs. Wear tracks at increasing magnification (**a**,**c**,**e**) for the Ti20–AlSi sample, and (**b**,**d**,**f**) for the Ti80–AlSi sample. (**c**,**d**) refer to the wear track edge, (**e**,**f**) refer to the middle of the wear track. (**a**,**b**) and the left micrographs of (**c**–**f**) are from the backscattered electrons detector; the secondary electron detector acquired the remaining micrographs.

SEM images of the overall (Figure 4a,b), lateral (Figure 4c,d), and central (Figure 4e,f) portions of the wear tracks can help us understand the wear mechanisms for the two materials dry sliding against cemented carbide balls. From the top-view, the wear tracks have different widths following the higher wear rate for the Ti80–AlSi; this is because the ball-ended pin penetrates deeper into the track after the increasing wear, and due to the spherical pin-end shape, the wear track widens (Figure 4a,b). A higher magnification at the edges of the track (Figure 4c,d) reveals the presence of fragments in both of them. Nevertheless, a higher degree of ductility and the formation of a thicker transferred layer on the surface of the Ti20–AlSi track are compatible with thicker side-ridges (Figure 4c,d).

Fragments with tiny features and larger wear particles are observed on both tracks, while abrasion and plowing marks are preferentially observable on the Ti20–AlSi track. The surface of the Ti20–AlSi sample is more worn than the Ti80–AlSi; this is attributed to adhesion, extrusion, and fragmentation caused by the different degrees of elastic–plastic deformation of the two metal–metal composites.

**Figure 5:**
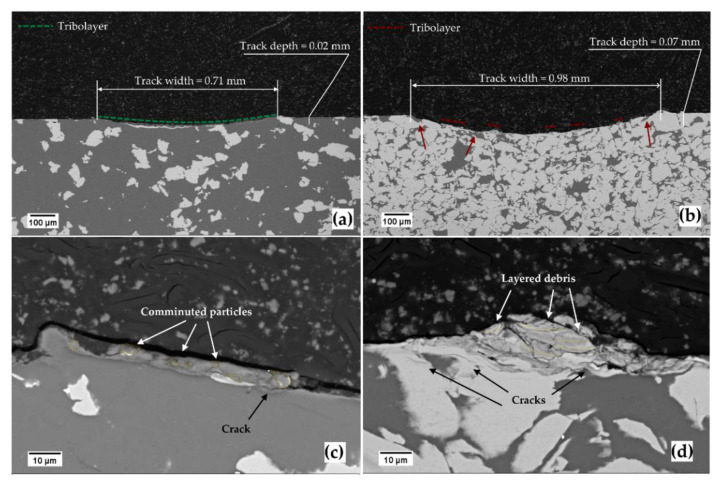
SEM cross-section micrographs of the wear tracks at increasing magnification. (**a**,**c**) for the Ti20–AlSi sample, (**b**,**d**) for the Ti80–AlSi sample.

From the cross-sectional observation of the wear tracks (Figure 5), more clues are delivered to understand the wear mechanism better. For example, the tribolayer formed on the Ti20–AlSi track is continuous along the whole wear track (green dotted line in Figure 5a), while the isolated layers for the Ti80–AlSi are mainly located over Ti particles (red dotted lines in Figure 5b). This is because the AlSi10Mg softer domains (red arrows) seem solicited by continuous abrasion and adhesion, leading to the exposure of Ti particles whose surroundings were depleted of the softer AlSi10Mg matrix.

The small portion of the tribolayer shown in Figure 5c covering the Ti20–AlSi wear track has a quasi-constant thickness and a compact layer of comminuted particles. This evidence suggests a mechanism of indirect tribo-oxidation.

Conversely, in the Ti80–AlSi cross-section (Figure 5d), the transferred material is localized on the Ti particles, with a layered structure that favors cracks nucleation. As a result, cracks propagate inside the layers of transferred material and under the surface of the fresh material, leading to a competing mechanism between tribolayer removal or material removal (i.e., wear).

**Figure 6:**
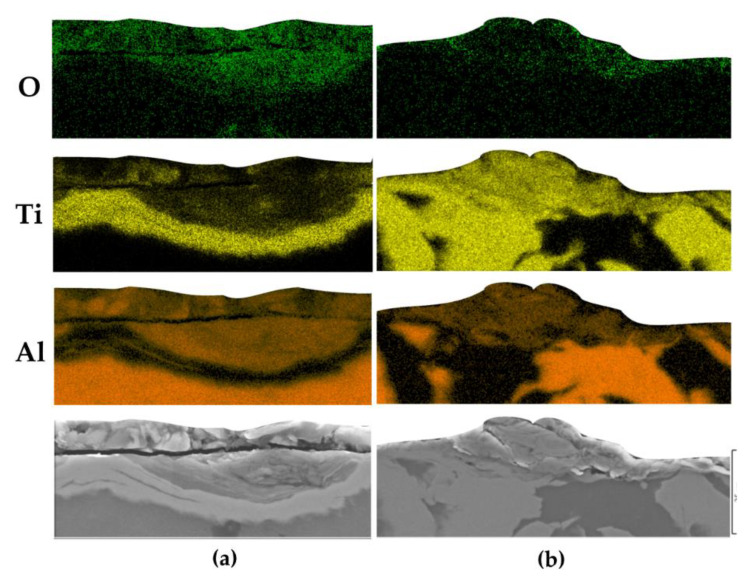
EDS maps of the tribolayer in cross-section. (**a**) Ti20–AlSi, (**b**) Ti80–AlSi.

Further evidence favoring a tribo-oxidation mechanism that reduces slide wear for the Ti20–AlSi sample comes from the EDS maps reported in Figure 6. The layer adhering on the surface in Figure 6a is mainly composed of aluminum, with a significant intensity of Oxygen; Titanium is present in minor traces, with some particles incorporated in the tribolayer, while the Titanium band in Figure 6a still belongs to the fresh material.

Conversely, the adhered asperity on the surface of the Ti80–AlSi sample in Figure 6b is mainly composed of Titanium. The mild intensity of the Oxygen map suggests a low degree of oxidation of the adhered asperity, not sufficient to generate a protective tribo-oxidative layer.

#### 3.1.3. Hertzian Stress Profile

**Figure 7:**
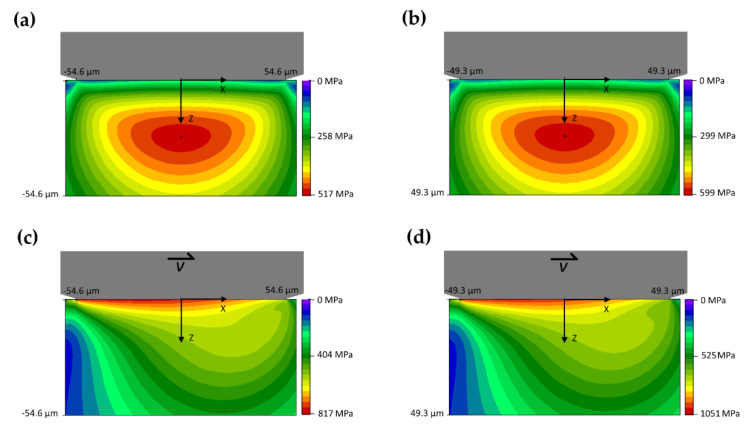
Two-dimensional Von Mises stress for the pin/disc non-conformal contact. (**a**,**c**) represent the WC–6Co/Ti20–AlSi contact in static (**a**) and dynamic (**c**) conditions, while (**b**,**d**) represent the WC–6Co/Ti80–AlSi contact in static (**b**) and dynamic (**d**) conditions.

The calculation of the Hertzian stress profiles further supports the wear mechanisms proposed, despite being representative of the first moments of the tribological test. Even if the applied load in the ball-on-disc tests is normal to the surface, different tensile and shear stresses arise in the contact plane and the subsurficial layers, as described in the Hertz theory [28]. Figure 7 describes the 2D stress profiles in the subsurficial layers for the two samples (Ti20–AlSi or Ti80–AlSi), both in static (Figure 7a,b), and dynamic (Figure 7c,d) loading conditions; the Von Mises stresses are reported as the criterion for the maximum equivalent stress.

The upper part of each graph schematically represents the pin (grey body), while the stresses are color-coded in the lower body, representing the disc under the contact area. It is known from the Hertz theory that *τ*_zx_ is the most critical stress component in static loading conditions, contributing for the most significant part to the Von Mises (VM) equivalent stress profile. The maximum VM stress is located under the contact surface and may cause plastic deformation if the material’s yield strength is exceeded.

The maximum VM stress for the two materials statically loaded under a 1.5 mm radius WC–6Co pin is 517 MPa at 26.8 µm depth for the Ti20–AlSi and 599 MPa at 24.9 µm depth for Ti80–AlSi (Figure 7a,b). The difference between the maximum equivalent stresses in the two materials is subtle but induces plasticization and possibly crack nucleation in the AlSi10Mg phase. This condition occurs when the 5 N loaded ball-ended pin touches the sample’s surface before starting the tribological test.

When the disc-shaped sample starts rotating, frictional forces develop on the contact plane, drastically modifying the subsurficial stress profile (Figure 7c,d). As a result, the maximum stress is no longer located below the surface but on the contact plane, and its absolute value increases significantly (817 MPa for the Ti20–AlSi and 1051 MPa for the Ti80–AlSi), damaging the surface. This condition occurs when the disc-shaped sample starts rotating under the 5 N loaded ball-ended pin, but it is no longer valid when the shape of the two surfaces deviates from the original sphere-on-flat geometry. Consequently, the wear track is progressively formed, and the contact between the two bodies becomes conformal, thus invalidating one of the fundamental assumptions of the Hertz theory.

Some similarities were found in the literature between the metal–metal composite materials presented in this study and the dry-sliding behavior of Aluminum or Titanium as the tribolayer formation regards.

The roles of sliding speed and load are crucial to determine the protective characteristic of the aluminum oxide formed. For example, in [29], Kim et al. observe that for pure aluminum sliding against an AISI 440 steel, high sliding speeds hinder the formation of a protective tribolayer while a load as high as 2 N is enough to increase oxidation. In that study, no pin-disc interaction was observed, with the material univocally transferred from the softer to the harder material. Furthermore, as regards sliding speed, the authors observed that a higher sliding speed (7.5 cm/s) also promoted a reduction in the coefficient of friction due to the formation of a protective oxide layer.

The sliding parameters investigated in the present work combine a higher load than that of Kim (5 N vs. 2 N) applied on a thinner pin, thus resulting in higher contact pressure responsible for the local rise in temperature.

High loads and high sliding speeds generate more heat than low loads and low sliding speeds. The increase in temperature reduces the local shear strength, resulting in the reduction of the friction coefficient.

This mechanism realistically describes the behavior observed on the Ti20–AlSi metal–metal composite, where the high fraction of AlSi10Mg promotes the formation of a tribo-oxidative regime.

For the Ti80–AlSi composite, the AlSi10Mg fraction is significantly lower, which cannot contribute to the formation of a continuous protective layer. The other metal responsible for forming a protective layer is Titanium, whose transferred amount in the contact is significantly predominant. However, pure Titanium is known for its weak tribological properties [30,31], limiting its application, especially when friction and wear are involved. The two main factors suggested as responsible for this behavior are the low resistance to plastic shearing and low work hardening (related to the high interplanar distance of its hexagonal close-packed (hcp) lattice), and the poor protective ability of the surface oxide that may form after the friction-related high local temperature rise. This characteristic results in the formation of comminuted wear debris, leading to a discontinuous and fragile tribolayer that is periodically removed so that wear can develop further.

### 3.2. Electrochemical Characterization

**Figure 8:**
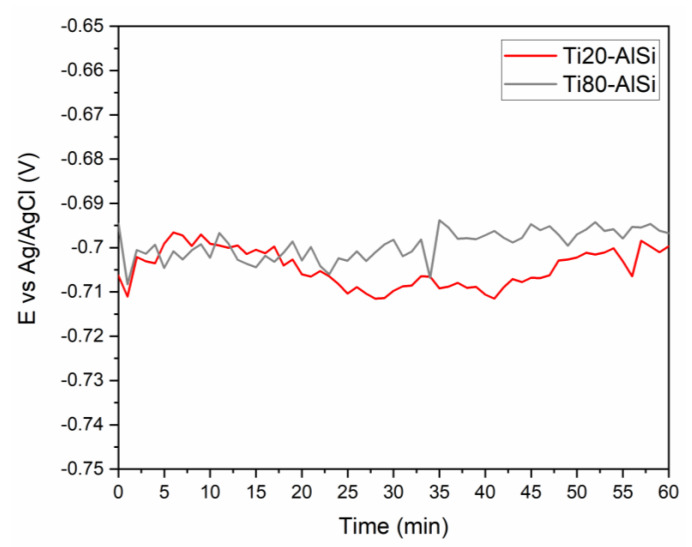
The open-circuit potential trend over time for the Ti80–AlSi (red line) and Ti80–AlSi (grey line) samples.

Figure 8 shows the results obtained by recording the OCP for one hour after immersing the samples in the test solution. The samples exhibited similar potentials oscillating by ±15 mV throughout the test period, indicating the system’s difficulty reaching equilibrium due to the presence of chloride ions that inhibit the formation of a stable passive layer.

The OCP range was in agreement with data referring to aluminum alloys in the same solution [32], which are generally more anodic compared with the potentials of titanium alloys under the corresponding conditions [33].

This evidence already underlines the predominance of aluminum in the corrosion process under steady-state conditions, as it is the most anodic species of the system studied. In addition, the higher amount of Ti resulted in a slightly higher initial potential for Ti80–AlSi.

During the first hour of immersion, the potential of both samples increased, as is typical for shell materials that tend to form a passive protective layer on their surface, shifting their potential towards more cathodic values [34]. As a result of the increasing trends, the OCP of sample Ti80–AlSi remained higher (−0.696 vs. Ag/AgCl) than Ti20–AlSi (−0.701 vs. Ag/AgCl), still for the higher amount of Titanium shifting the potential to more cathodic values.

**Figure 9:**
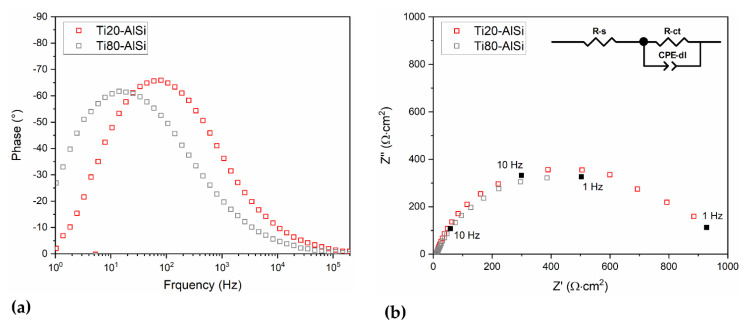
Electrochemical Impedance Spectroscopy was measured at OCP for the Ti20–AlSi (red squares) and Ti80–AlSi (grey squares) samples. (**a**) Bode plot of phase angle; (**b**) Nyquist plot, the inset shows the equivalent electric circuit used for fitting the measured data.

Impedance measurements were carried out to investigate the properties of the sample/solution interface. This technique can detect and quantify frequency-dependent processes by examining their time constants, *τ*, defined as τ=R · C, where R and C are the resistance and capacitance associated with the electrochemical process. The analysis of the Bode Plot (Figure 9a), which reports the phase distribution for both samples along the frequency coordinate, allows identifying a single process, indicated by a peak with *ϕ* = −62° at 10 Hz for Ti80–AlSi, and *ϕ* = −66° at 60 Hz for Ti20–AlSi. This feature indicates the presence of a non-ideal capacitive contribution for both samples, as a pure capacitor would exhibit a *ϕ* = −90°. Additionally, the different frequencies of the peak indicate a faster process for the Ti20–AlSi sample. At higher frequencies, the phase angle converges towards 0° for both samples, indicating the presence of a pure resistive contribution.

The spectra plotted in the Nyquist diagram (Figure 9b) confirmed the presence of one time constant for each sample, represented by a single semicircle with the center below the x-axis. This characteristic indicates an R//C-like behavior with a non-ideal capacitance and confirms the interpretation of the phase angle plot. In systems such as the one considered here, the non-ideal capacitance can be described by a Constant Phase Element (CPE) [35]. The impedance spectra analysis led to propose an equivalent electric circuit used to fit the data, consisting of a resistor (R-s) in series with a R//CPE mesh (R-ct//CPE-dl), as shown in the inset of Figure 9b. R-s describes the solution’s resistance, while R-ct is associated with charge transfer and CPE-dl with an electrical double layer. This association was obtained from values of the effective double-layer capacitance (Ceff-dl, Table 4) calculated from the CPE parameters, resulting in the range between 10 and 100 µF · cm^−2^ [36,37]. This calculation was performed using Brug’s equation [38], assuming that the measured non-ideal capacitive behavior is due to surface inhomogeneities of the sample [39]:

(2) $$C_{\mathrm{eff}}{=Q}^{\frac{1}{n}}{\left(\frac{1}{R_{s}}+\frac{1}{R_{\mathrm{ct}}}\right)}^{\left(\frac{n-1}{n}\right)}$$

where Q and n are the C.P.E. parameters.

The absence of a second process at lower frequencies indicates the lack of formation of a passive film on the samples. This behavior is due to chloride ions that, even at neutral pH, prevent passivation and lead to soluble chloride complexes, which diffuse into solution according to Reaction 1 (see Equation (3)) [40].

(3) $${\mathrm{Al}}^{3+}{+2\mathrm{Cl}}^{-}{+2\mathrm{OH}}^{-}\to \mathrm{Al}{\left(\mathrm{OH}\right)}_{2}{\mathrm{Cl}}_{2}^{-}$$

**Figure 10:**
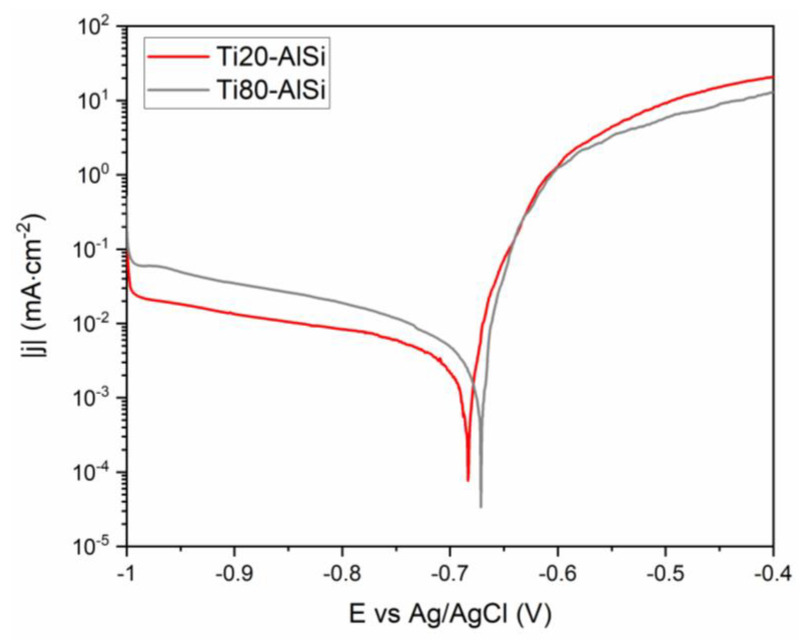
Polarization curves measured between −1 V vs. Ag/AgCl and −0.4 V vs. Ag/AgCl with a scan rate of 0.5 mV s^−1^.

Figure 10 shows the polarization curves of the two samples measured from the cathodic to the anodic potential limits. The current densities of the cathodic regions gave lower values than those of the anodic; this indicates that in such systems, corrosion is limited by the cathodic reaction of oxygen reduction:

(4) $${2H}_{2}{O+O}_{2}{+4e}^{-}\to {\;4\mathrm{OH}}^{-}$$

The measured cathodic current of the Ti80–AlSi sample resulted higher than Ti20–AlSi, in agreement with the higher Titanium content facilitating the cathodic reaction.

Near the OCP, the measured current followed linear trends, indicating a region of Tafel behavior where the driving force of the reaction is charge transfer. This potential range was used to calculate the kinetic parameters shown in Table 5, which confirm the highly cathodic behavior for the Ti80–AlSi sample, as indicated by the OCP and EIS results. Additionally, the resulting polarization resistances, Rp, were higher than those calculated from EIS data but confirmed the better corrosion resistance of the sample Ti80–AlSi.

The anodic branch of the polarization curve showed an active behavior due to the absence of a passive layer protecting the whole sample. The surface of the Titanium reaction 3 (Equation (5)) [33], which is stable at the pH of the test solution and throughout the potential range studied [41].

(5) $${\mathrm{Ti}+2O}^{2-}\to {\mathrm{TiO}}_{2}{+4e}^{-}$$

However, the difficulty of aluminum alloy to form a passive layer in the presence of chloride ions exposes unprotected regions to the solution where the dissolution reaction takes place (Reaction 1, Equation (3)), leading to an increase in anodic currents. The lower Aluminum alloy content in the Ti80–AlSi sample resulted in lower anodic current density, confirming its role in corrosion with the dissolution process. The measured pH increased to 7.6 and 7.9 for the Ti20–AlSi and Ti80–AlSi, respectively, confirming the formation of alkaline species in the solution after anodic polarization.

#### 3.2.1. SEM-EDS Analysis

**Figure 11:**
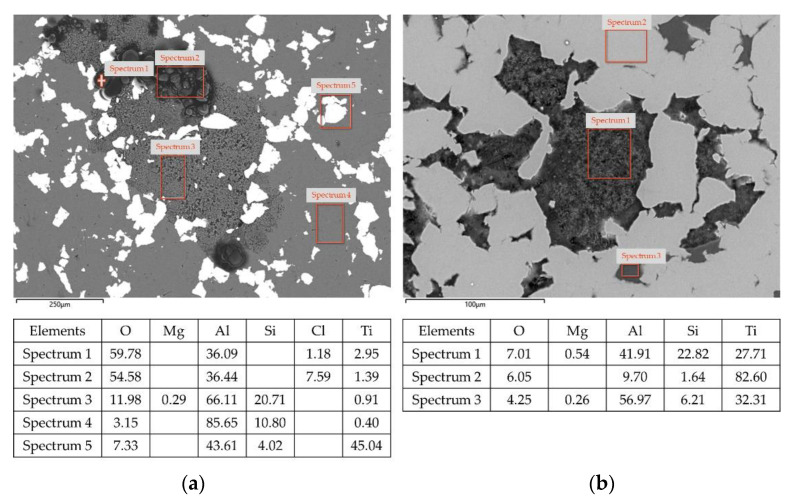
SEM images. EDS analysis in different regions of the specimen and relative quantitative composition in weight percentage. (**a**) Ti20Al80 sample; (**b**) Ti80A20 sample.

SEM-EDS analysis confirms the results of the electrochemical measurements, with AlSi10Mg domains being more susceptible to corrosion. The composition of the AlSi10Mg domains in the Ti20–AlSi sample is shown in Figure 11a and reveals the presence of O, Al, Mg, Si, Cl, and Ti. Furthermore, three distinct regions characterize the sample surface after the effect of corrosion: region 1 (spectrum 1 and spectrum 2) is covered by chlorine-based corrosion products; region 2 (spectrum 3) is characterized by a spongy morphology where deep surface oxidation is detected; and region 3 (spectrum 4 and spectrum 5) where no corrosion occurs. The EDS analysis for the selected areas highlights severe oxidation and the presence of Cl compounds in the AlSi10Mg domains: such evidence further supports the hypothesis regarding the poor passivating effect of the Al_2_O_3_ oxide film, causing a localized corrosion process limited to the AlSi10Mg areas.

The corrosive process is restricted to a more confined area regarding the Ti80–AlSi corroded microstructure (Figure 11b). The AlSi10Mg domains are still the preferential sites for corrosion, but due to the lower volume of Al-based alloy in the material, the corroded area is more limited overall. In this case, Cl was not found from the EDS analysis of the corroded areas, probably due to the short amount of material involved in the corrosion process.

#### 3.2.2. Raman Spectra

After electrochemical tests and SEM analysis, the chemical species formed after corrosion was accurately identified by Raman spectroscopy for both materials. The Raman spectra and the observed Raman shifts from the analyzed areas are shown in Figure 8. The investigated regions of interest are: (1) the corroded Al domains, (2) lightly-corroded aluminum (sponge-like areas), and (3) the Titanium domains. Spongy morphology is the Al alloy as Figure 11a spectrum 3.

**Figure 12:**
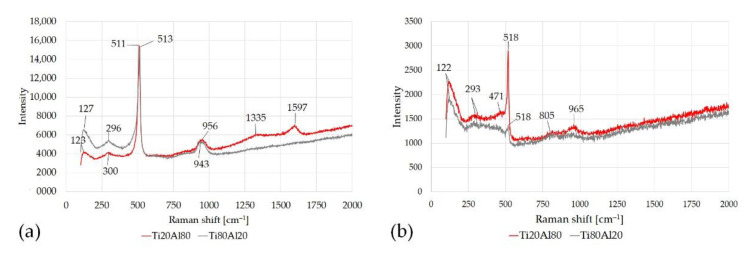
Raman shift for different areas in specimens Ti20–AlSi and Ti80–AlSi. (**a**) the corroded Aluminum domains, (**b**) lightly-corroded aluminum.

Figure 12 shows Raman spectra observed in Al domains, along with the measured Raman shift observed. A similar Raman shift characterizes both the Raman spectra of the corroded Al (Figure 12a) and the lightly-corroded Al (Figure 12b). Peaks associated at the Raman shifts 122, 123, 127 cm^−1^, from literature, regarding the products of corrosion Al–Cl based [42].

Silicon is characterized by a well-known specific peak at 520 cm^−1^ and a secondary peak of about 950 cm^−1^ [43,44]. Both peaks were found in Al corroded and lightly-corroded Al areas. In addition, in the lightly-corroded Al area of sample Ti20–AlSi, a weak peak of 471 cm^−1^, compatible with Mg_2_Si, is detected [45]. Further peaks at 1335, 1590 cm^−1^ were detected; from literature, they may correspond to minor carbon phases [44].

**Figure 13:**
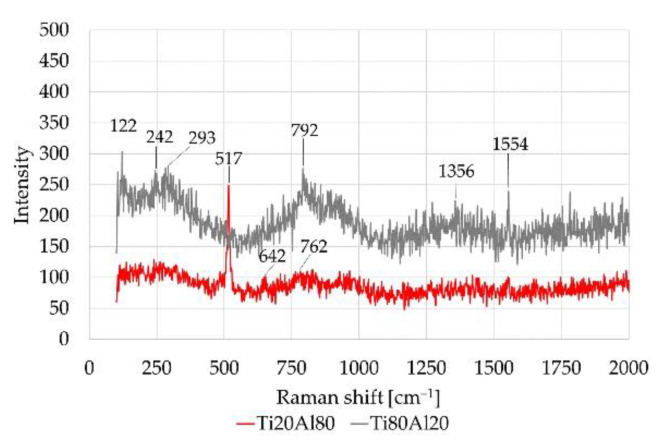
Raman shift for different Titanium areas in specimens Ti20–AlSi and Ti80–AlSi.

As for Titanium domains (Figure 13), Peaks associated with corrosion Al-, Cl-based products were not found in the Ti area of specimen Ti80–AlSi. Conversely, a very weak peak at 242 cm^−1^ was detected in the Ti area of sample Ti80–AlSi and could be associated with the AlCl_3_ species, as reported in [42].

A weak peak of Corundum Al_2_O_3_ was found near the Ti phases in Ti20–AlSi samples at 642 cm^−1^ [46]. Electrochemical tests highlighted that Al_2_O_3_ on the AlSi10 areas is not stable during the corrosion tests due to the presence of chloride species and, after its breakdown, is unable to re-form itself. Conversely, Ti oxide TiO_2_, especially in the Ti80–AlSi specimen, was not affected by the corrosion tests. Electrochemical tests highlight a cathodic behavior for the Ti80–AlSi specimen. Thus, TiO_2_ oxide was detected at the Raman shift near 750–800 cm^−1^. The TiO_2_ peaks may slightly change Raman shift value depending on the oxide type (anatase, rutile) [47,48]. As observed in the corroded Aluminum (Figure 12a), peaks at 1356, 1554 cm^−1^ were detected; from literature, they may correspond to minor carbon phases or O_2_ [44].

Table 6 summarizes the Raman shifts observed in samples Ti20Al80 and Ti80Al20, along with their indexing.

## 4. Conclusions

This study presents wear and corrosion characterization of two Ti–AlSi10Mg metal–metal composites obtained via a field-assisted rapid sintering technique called electro-sinter-forging. Several studies concerning Ti–Al metal–metal composites are found in the literature, being that this system is the most studied among metal–metal composite materials, but wear and corrosion characterizations were not presented so far.

From wear characterization in dry-sliding conditions against a WC–6Co pin, the following conclusions are derived:

- The metal–metal composite with 20% Ti and 80% AlSi10Mg has a higher wear resistance than the 80% Ti–20% AlSi10Mg due to a protective oxide layer formed by the AlSi10Mg phase: such layer switches the wear mechanism from adhesive/abrasive to tribo-oxidative. This conclusion is confirmed by the differences in wear rate and observing the wear tracks.
- The metal–metal composite’s wear mechanism with 80% Ti and 20% AlSi10Mg is a mix of abrasive and adhesive wear. The protective layer formation is hindered in these sliding conditions because Titanium, due to its low resistance to plastic shearing and low work hardening, forms comminuted wear debris without building a continuous layer on the wear track.

From the electrochemical characterization and the analysis of the corroded surfaces, the following conclusions are drawn:

- A higher amount of Titanium in the samples increases the OCP due to the cathodic behavior related to forming a stable, protective TiO_2_ layer.
- In the presence of chloride ions, the formation of a passive layer on the Aluminum alloy portions of the samples is hindered, thus exposing unprotected regions to the solution, where the dissolution occurs. In addition, the EDS analysis highlighted severe oxidation and the presence of Cl-based compounds on AlSi10 areas where localized corrosion processes take place.
- The Raman spectra confirmed the evidence from the electrochemical measurements by detecting only traces of Al_2_O_3_ and significant amounts of AlCl-based compounds in correspondence of anodic zones; TiO_2_ was detected in Ti domains where the cathodic reaction occurs.

## Figures and Tables

**Table 1:** Chemical composition of the powder used for the fabrication of specimens in weight percentage.

Powder	Al	Si	Mg	Ti	Fe	C	O	N	H
AlSi10Mg	Bal.	10.20 ± 0.13	0.35 ± 0.07	-	-	-	<0.09	<0.01	<0.007
Ti-Grade 2	-	-	-	Bal.	0.30 ± 0.02	0.08 ± 0.02	<0.25	<0.03	<0.015

**Table 2:** Pin-on-disc test parameters.

Ball Diameter (mm)	Ball Material	Sliding Speed (m/s)	Sliding Distance (m)	Applied Load (N)	Surface Roughness, Ra (µm)
3	WC-6Co	0.02	100	5	0.4 µm

**Table 3:** Weight percentage compositional semi-quantitative analysis after EDS for the tribolayers transferred on the WC–6Co pins.

	O	Al	Si	Ti
EDS_1	31.61	48.05	4.98	15.36
EDS_2	15.73	15.23	1.97	67.07
EDS_3	10.14	19.30	2.34	68.22

**Table 4:** EIS fitting results with attributions: s = solution; ct = charge transfer; dl = double layer.

	R-s/Ω·cm^2^	R-ct/Ω·cm^2^	CPE-dl-Q/Ω^−1^·cm^−2^·sn^−1^	CPE-n	Ceff-dl/F·cm^−2^
Ti20–AlSi	10.2	921.50	6.23 × 10^−5^	0.86	1.7 × 10^−5^
Ti80–AlSi	10.1	926.50	2.54 × 10^−4^	0.81	4.2 × 10^−5^

**Table 5:** Kinetic values obtained from Tafel fit.

	I_0_/A·cm^−2^	Ba/mV·dec^−1^	Bc/mV·dec^−1^	Rp/Ω·cm^−2^
Ti20–AlSi	5.89 × 10^−5^	14	47	1285
Ti80–AlSi	4.89 × 10^−5^	13	67	1546

**Table 6:** Raman shift measured for (a) corroded aluminium; (b) spongy-like aluminium; (c) Titanium.

Raman Shift Indexing Ti20–AlSi [cm^−1^]			
Species	Corroded Al	Lightly-corroded Al	Titanium
AlCl_4_^−^ *^,[a]^	123	122	122
Al	300	293	-
Mg_2_Si	-	471	-
Si	511, 956	518, 965	517, 965
Al_2_O_3_	-	-	642
TiO_2_	-	-	762
Minor carbon phases [44] *^,[b]^	1335	-	-
Carbon phase broadening or O2 [44] *^,[b]^	1597	-	1554
Raman shift indexing Ti80-AlSi [cm^−1^]			
Species	Corroded Al	Lightly-corroded Al	Titanium
AlCl_4_^−^ *^,[a]^	127	122	-
Al	296	293	293
AlCl_3_ *^,[a]^	-	-	242
Si	513, 943	518, 965	-
TiO_2_	-	805	792
Minor carbon phases *^,[b]^	-	-	1356
Carbon phase broadening *^,[b]^	-	-	1554

* hypothetical peak designation according to literature. [a]: [42]; [b]: [44].
